# Perceptions of Customer Incivility, Job Satisfaction, Supervisor Support, and Participative Climate: A Multi-Level Approach

**DOI:** 10.3389/fpsyg.2021.713953

**Published:** 2021-10-13

**Authors:** Zselyke Pap, Delia Vîrgă, Guy Notelaers

**Affiliations:** ^1^Department of Psychology, West University of Timişoara, Timişoara, Romania; ^2^Department of Psychosocial Science, University of Bergen, Bergen, Norway

**Keywords:** participative climate, job satisfaction, supervisor support, customer incivility, multi-level, job resources, job demands

## Abstract

Perceived customer incivility can be a significant day-to-day demand that affects frontline service employees’ job satisfaction. The current research focuses on job resources on multiple levels that serve as buffers in the face of this demand. We tested a multi-level model in which supervisor support (at the employee level) and participative climate (at the work-unit level) moderate the negative relationship between perceived customer incivility and job satisfaction. We used multi-level analysis with self-reported cross-sectional data collected from 934 employees nested in 107 work units of a large clothing shop chain in Belgium. The results showed that both supervisor support and participative climate moderate the negative relationship between perceived customer incivility and job satisfaction. The theoretical contribution of this study resides in an extension of the JD-R theory to simultaneously conceptualize resources on multiple levels. In the meantime, we focus on practical, hands-on resources that organizations can implement to protect service employees from the adverse effects of perceived customer incivility.

## Introduction

The frequent interactions inherent to front-line employees’ jobs often include dealing with customers who behave in unfriendly and disrespectful ways (Han et al., 2016). This represents a daily hassle for retail workers, and since “the customer is always right” policies are characteristic of organizations that thrive on customer satisfaction (Wilson and Holmvall, 2013), front-line employees need to frequently regulate their emotions and spend resources to offer service with a smile to every customer (Koopmann et al., 2015). *Customer incivility* is defined as the employee’s perception that the customer is behaving in an uncivil manner (e.g., being disrespectful or insulting; van Jaarsveld et al., 2010) and has been negatively related to employee well-being and job satisfaction (Alola et al., 2019). While a plethora of research has shown that it has adverse effects in terms of increasing employees’ stress levels (Kim et al., 2014), leading to emotional exhaustion (van Jaarsveld et al., 2010), in a domain where customer satisfaction is a central preoccupation for organizations, employee satisfaction also represents a primary outcome due to its’ consistent relationship to customer service quality and customer satisfaction (Brown and Lam, 2008). Since interactions with the customer represent the main activity of a front-line retail employee and eliminating this stressor is not a straightforward option, we focus on finding moderators in the workplace dynamics with supervisors and colleagues (Yang and Lau, 2019).

This study is guided by the postulates of Job Demands-Resources Theory (JD-R; Bakker and Demerouti, 2017), which explains work-related outcomes like satisfaction, based on two main processes (a motivational and a health impairment process), which are a function of two key elements: job demands and resources. Central to JD-R and the present study are resources, or those physical, psychological, social, and organizational aspects of the job that motivate employees by being functional in achieving work goals, stimulating growth and development, and reducing job demands, and associated costs (Bakker and Demerouti, 2017). This definition has some theoretical limitations with implications for the design of this study and the theoretical reasoning behind our hypotheses.

First, it implies a functional equivalence of resources at all levels (be they physical, psychological, social, or organizational). It postulates that they work the same way at and across all levels (Bakker and Demerouti, 2017). However, aggregating individually perceived resources to the group level makes them phenomenologically distinct (Bliese, 2000). Transferring inferences derived from individual-level research to other organizational levels is a fallacy that can lead us to biased inferences (Kozlowski and Klein, 2000). Hence, while we can build on valuable individual-level evidence suggesting their existence and effects (Chen et al., 2021), we need to be cautious in naturally accepting that resources at higher levels work the same way they do at the individual level. To construct unbiased knowledge about multi-level phenomena in organizations, constructs need to be measured and theorized at the level they reside at Kozlowski and Klein (2000). Since theoretical reasoning for cross-level relationships within the JD-R tradition is based on a research base that for a very long time has been focused on the individual level (Bakker and Demerouti, 2017), much more evidence is needed to draw strong conclusions when it comes to cross-level moderating effects (Jex et al., 2014).

Second, the JD-R theory postulates a skeleton of various relationships but is not very specific in explaining the psychological mechanisms behind effects (Bakker and Demerouti, 2017). Consequently, researchers usually borrow from complementary theories that can help construct more in-depth theoretical reasoning in JD-R studies (Schaufeli and Taris, 2014). Similar to previous work, we also rely on postulates from *Conservation of Resources Theory* (COR; Hobfoll, 2011) and *Self-Determination Theory* (SDT, Gagné and Deci, 2005; Deci et al., 2017). According to SDT, resources have their motivational potential postulated in JD-R theory when they satisfy employees’ basic needs for autonomy, competence, and relatedness (Deci et al., 2017; Nielsen et al., 2017). When employees perceive the work context as one that provides the necessary resources to satisfy these needs, they tend to develop internalized forms of motivation, be more performant in challenging tasks, and form more positive attitudes toward their jobs (Gagné and Deci, 2005). Chen et al. (2021) have argued that empowering HRM practices could represent an organization-level characteristic that buffers the impact of customer incivility on basic needs’ satisfaction. These results, however, are still waiting for confirmation at higher levels of analysis that can differentiate between the individual impact of perceptions of the environment, and the actual impact of the environment, which is more than the sum of individual perceptions (Bliese, 2000; Kozlowski and Klein, 2000).

Conservation of Resources Theory theory presents another valuable theoretical reasoning for the possible cross-level moderating effects under the concept of *resource caravan passageways* (Hobfoll, 2011). These are environmental conditions offered by the organization that support, foster, enrich and protect the resources that individuals can access to cope with everyday difficulties (Hobfoll et al., 2018). From this point of view, employees can draw on these contextual resources to replenish resources that were depleted in interactions with uncivil customers (Koopmann et al., 2015; Wang and Wang, 2017). Hence, both COR and SDT theories add to the explanatory power of JD-R by elucidating processes through which higher-level resources could minimize or recover resource loss (Schaufeli and Taris, 2014; Boukis et al., 2020).

**FIGURE 1:**
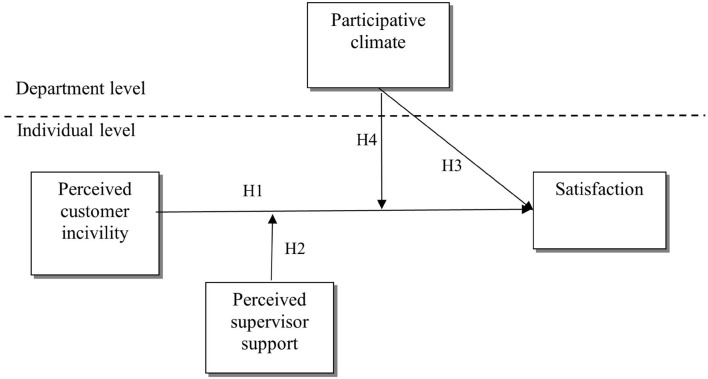
Amulti-level model of perceived customer incivility, satisfaction, supervisor support, and participative climate.

Based on this theoretical background, we propose a multi-level JD-R model (Figure 1) of perceived customer incivility (PCI) and *job satisfaction*, conceptualized as a pleasurable emotional state derived from one’s work (Brown and Lam, 2008). We argue that *supervisor support*, defined as the extent to which employees believe that their supervisors appreciate, value, and care about their well-being (Zhu et al., 2019), is an employee-level resource that can alleviate the adverse effects that PCI has on job satisfaction (Alola et al., 2019). Further, we propose *participative climate* as a shared, group-level resource that moderates the relationship between PCI and satisfaction beyond the effects of supervisor support. In this context, participative climate constitutes a resource caravan passageway that employees can turn to and replenish resources handling negative interactions with customers better (Hobfoll et al., 2018).

From a theoretical point of view, this research extends existing knowledge through the conceptualization of participation as a shared, group-level resource in the form of climate (Füllemann et al., 2016; Pap et al., 2020). Previous studies have advocated for the conceptualization of shared, group-level resources as climate in service-employee contexts (Füllemann et al., 2016) since various types of unit-climate have significant direct or buffering effects on employee subjective well-being akin to the effects of job resources (Carr et al., 2003). Hence, while there is a theoretical and empirical basis that warrants attention to these kinds of multi-level relationships (Bakker and Demerouti, 2017, 2018), this is the first study that considers participative climate as a group-level moderator in the PCI – satisfaction relationship alongside to supervisor support received at the individual level. Although a long-standing research stream indicates that all organizational phenomena are nested in a higher-level context, which often has a direct or moderating effect on lower-level outcomes (Kozlowski and Klein, 2000; Jex et al., 2014), the vast majority of studies investigating employee well-being focus on the physical and psychological level of the individual, leaving a gap around empirical evidence supporting the proposed interaction effects across levels (Bakker and Demerouti, 2017).

Multi-level theoretical frameworks of employee well-being are still relatively new and are in need of more evidence that sustains their practical relevance in organizations (Bakker and Demerouti, 2018; Hobfoll et al., 2018). Thus, our work contributes to existing knowledge by answering the call of Bakker and Demerouti (2018) to investigate the effects of demands and resources that span organizational levels in order to develop more effective interventions. Hence, from a practical point of view, this research is valuable due to the focus on hands-on, concrete resources that managers and organizations can implement on two different but complementary levels in their attempts to help employees better cope with uncivil customers (Nielsen et al., 2017). In a literature stream that focuses mainly on the customer-employee dyad in the attempts to understand the adverse effects of customer incivility (Yang and Lau, 2019), empirical evidence that bridges multiple levels of the organizational knowledge together (employee reactions, supervisor effects, and collective resources in the work unit) can constitute significant scientific advantage (Kozlowski et al., 2013).

### Perceptions of Customer Incivility and Job Satisfaction

Perceptions of customer mistreatment have been linked to significant adverse effects on employees’ job satisfaction and well-being (for a review, see Koopmann et al., 2015). In the JD-R perspective, PCI represents a job demand (Boukis et al., 2020), which causes strain and adverse employee outcomes through a health impairment process in which employees’ emotional and instrumental resources are drained (Bakker and Demerouti, 2018). In line with this postulate, interactions with uncivil customers have been shown to significantly increase employee’s stress levels (Kern and Grandey, 2009; Kim et al., 2014), leading to emotional exhaustion (van Jaarsveld et al., 2010), and decreased satisfaction (Alola et al., 2019). Hence, perceived customer incivility is an essential demand that fuels the health impairment process, generating high levels of stress. In addition, the front-line employee can interpret customer incivility as an indicator of goal failure and lack of performance (Koopmann et al., 2015), thereby thwarting the satisfaction of basic psychological needs for competence and autonomy (Gagné and Deci, 2005; Deci et al., 2017), and decreasing satisfaction (Kim et al., 2014; Chen et al., 2021). While this relationship was primarily studied in health care settings (Wang and Wang, 2017), restaurants (Han et al., 2016), and front-line employees in hotels and resorts (Yang and Lau, 2019), there is also empirical evidence showing a negative link between PCI and job satisfaction among retail employees (Kern and Grandey, 2009; Wilson and Holmvall, 2013).

*H1*.*: Perceived customer incivility is negatively related to job satisfaction*.

### Supervisor Support as a Moderator

Extensive literature indicates that certain leadership behaviors are highly effective in promoting employee job satisfaction (Wegge et al., 2010). We argue that beyond the evident direct effects that leaders can have on employee well-being, their support is also a key element in helping employees face difficult situations, such as interactions with rude and unfriendly customers (Han et al., 2016; Zhu et al., 2019). According to the buffering effect of job resources postulated in the JD-R theory, supervisor support can help reduce job demands and associated psychological and physiological costs (Bakker and Demerouti, 2018). Supportive supervisors, who provide guidance and training in employee’s customer service role, elicit better sales skills and service performance (Liaw et al., 2010). By this, supervisors are empowering the employee to take effective action when meeting the next customer, essentially satisfying the basic needs for autonomy and competence thwarted by PCI (Gagné and Deci, 2005; Chen et al., 2021) and restoring satisfaction (Pettijohn et al., 2007; Deci et al., 2017). Also, in line with COR theory (Hobfoll, 2011), a supportive supervisor, through broader access to organizational resources and greater decision-making authority, can offer the necessary aid to deal with the situation by restoring or replacing depleted emotional and instrumental resources (Hobfoll et al., 2018) that are lost due to the frequent negative interactions with uncivil customers (Koopmann et al., 2015; Boukis et al., 2020). Supervisor support has significantly moderated the effect of PCI on forms of well-being in a sample of restaurant employees (Han et al., 2016) and among employees of an integrated resort (Zhu et al., 2019). However, this moderating effect was not significant among hotel front-line employees (Karatepe, 2011), which warrants a further focus on the buffering role of supervisor support in different industries (Boukis et al., 2020).

*H2*.*: Supervisor support buffers the negative impact that perceived customer incivility has on job satisfaction*.

### Participative Climate

As part of the wider employee involvement and organizational leadership model (EIOL; Wegge et al., 2010), organizational participation has been defined through various processes whereby power, influence, decision-making, and responsibility are shared among employees, supervisors, and other relevant agents working in, or with the company. According to Tesluk et al.’ (1999) seminal article, participative climate captures employee perceptions of such employee involvement systems, signaling to employees that participation in work planning, decision making, and on-the-job problem solving are relevant organizational goals that are expected and rewarded practices within the organization (Schneider et al., 2011). Climate exerts top-down, cross-level effects on employee well-being (Kozlowski and Klein, 2000; Jex et al., 2014), but is created through a bottom-up process labeled as *emergence*, whereby dynamic interaction processes among employees yield phenomena that originate from the cognition, affect, and behaviors of individuals, and manifests on higher levels, in the form of climate (Kozlowski et al., 2013). Employees tend to develop a shared understanding of the environment, which facilitates the emergence of climate, reflecting a sense of shared meaning among co-workers (Jex et al., 2014). Employees need to frequently experience that the organization values their input in decisions regarding their work and openly communicates about these decisions (Seki et al., 2016), but also to have experiences of concrete, individual participation, a shared meaning of participation with beneficial effects to arise (Weber et al., 2020).

In the JD-R theory perspective, participation is a resource that can be beneficial to employees at the fastest route through being directly instrumental in achieving work goals or stimulating personal growth, learning, and development (Bakker and Demerouti, 2017; Nielsen et al., 2017). Working in a participative climate, employees also perceive that more control is offered to them in making decisions regarding customers, eliciting greater experience of responsibility and recognition (Bosak et al., 2017). When employees perceive that individuals have the power to influence decisions and participate in important discussions in their work unit, they feel that their work can involve action with a sense of volition and the experience of choice (Gagné and Deci, 2005). This, in turn, can elicit internalized forms of motivation through fulfilling the needs for autonomy and competence (Gagné and Deci, 2005; Deci et al., 2017) and giving rise to more positive attitudes, like job satisfaction (Miller and Monge, 1986; Bosak et al., 2017; Weber et al., 2020; Chen et al., 2021). Empirical evidence shows that perceptions of participative climate are better predictors of job satisfaction and performance than actual participation in specific decisions (Miller and Monge, 1986). A multi-level study showed that in work units where employees perceive the existence of an involvement climate and actively participate in decision-making, they find more satisfaction in their job (Bosak et al., 2017). Finally, Tesluk et al. (1999) showed that participative climate, both perceived individually and aggregated at the unit-level (but on the higher, district level), significantly predicted satisfaction in the relationships with co-workers and supervisors and with the attention and recognition they perceived for performing well at their jobs and making suggestions (measured as intrinsic satisfaction, Tesluk et al., 1999).

*H3*.*: Unit-level participation climate has a positive relationship with job satisfaction*.

Facing rude or disrespectful customers can be a daunting task, which according to COR theory, depletes individual resources and predisposes employees to loss cycles whereby individuals have fewer and fewer resources to face further losses (Koopmann et al., 2015). However, COR theory also states that the degree to which the employee has access to contextual resources in the social environment can play an important role in resource acquisition and protection from resource depletion (Hobfoll, 2011). Participative climate can constitute a resource caravan passageway (Hobfoll et al., 2018) because it could protect existing resources (e.g., knowing that one has a voice in defining procedures and rules in dealing with rude customers might preserve self-esteem and optimism), or it can generate new resources (e.g., a greater sense of autonomy and control over the situation; Castanheira and Chambel, 2010; Chen et al., 2021). The possibility of resource gain becomes salient in the context of resource loss (Hobfoll, 2011), meaning that a shared understanding that participation is accepted rewarded, and essential in the work unit becomes especially important in the context of PCI. In such environments, employees might feel that their experience and opinion in dealing with these interactions will be listened to and considered, eliciting ownership over the situation and building resilience (Hobfoll et al., 2018). Available opportunities to propose and implement better customer service strategies might motivate the employee to be actively involved in generating performant service solutions, which drive satisfaction (Pettijohn et al., 2007; Chen et al., 2021).

There is a relative lack of empirical evidence positioning participative climate as a buffer between PCI and satisfaction. One recent longitudinal study demonstrated that individual-level perceptions of empowerment HRM practices (including participation among other practices in one general measure) constitute a boundary condition for the relationship between customer mistreatment and employee satisfaction (Chen et al., 2021). In another individual-focused study, PCI related less positively to exhaustion in teams where employees had more participation opportunities (Hu et al., 2018). Based on the finding that it moderated the relationship between several job demands and symptoms of depression, participative climate has been advocated to be vital in creating healthy workplaces (Seki et al., 2016).

*H4*.*: Unit-level participation climate buffers the negative effect of perceived customer incivility on job satisfaction*.

## Materials and Methods

### Participants and Procedure

The data for this study was collected by a Monitoring and Statistical Consulting company from Belgium specialized in measuring occupational stress for Belgian Health and Safety Executives. Nine hundred thirty-four (*N* = 934) employees, nested in 107 work units of a large clothing shop chain, completed self-reported questionnaires. Cluster size varied from 2 to 24 members per unit with a mean of 8.7. No members of the surveyed organization had access to any of the completed questionnaires, herewith guaranteeing anonymity.

#### Sample Characteristics

The sample consisted of a large proportion of women (94.8%). 12.3% had a minimum of 25 years, 20.4% were situated in the range of 25–34 years, 17.1% between 35 and 44 years, 34% between 45 and 54 years, and 16.1% of participants had over 55 years. Most were part-time employees (64.4%) with a mean tenure of 14.3 years (SD = 12.3). Regarding education, 3.2% completed primary school, 82.4% had completed secondary school, and 14.3% had a Bachelor’s or higher degree. 62.4% of participants worked as shop assistants, about 10% occupied posts in visual merchandising, and 25.5% held managerial positions.

### Measures

Subscales of *The Short Inventory to Monitor Psychosocial Hazards* (SIMPH; Notelaers et al., 2007) were used to measure all study variables. The SIMPH was developed by Notelaers et al. as a theoretically driven and empirically solid instrument with the aim of monitoring psychological risks that employees are exposed to in organizations. It has become a very popular instrument to conduct psychosocial risk analysis in Belgium, providing a skeleton that is completed upon request by extra instruments aligned with the JD-R theory and the specific needs of different organizations. Beyond its’ extensive practical use by the statistical consulting company that collected the data for this study, a number of research articles have relied on it over the years (e.g., Schreurs et al., 2011; Notelaers et al., 2012; Pap et al., 2020).

*Perceptions of customer incivility* were measured with four items on a 4-point Likert scale from 0 = “never” to 3 = “always”. Questions referred to the frequency of interactions with unfriendly, verbally abusive clients (”How often do you have to deal with unfriendly clients?”). The scale had good internal consistency (α = 0.76).

*Satisfaction* was measured with five items on a dichotomous response scale (e.g., 0 = “yes”; 1 = “no”). Items, in general, referred to pleasure in work (”Mostly, I am pleased to start my day’s work.”) The reliability analysis revealed a good internal consistency for this scale (α = 0.72).

*Supervisor support* was measured by three items on a 4-point Likert scale from 0 = “never”, to 3 = “always”. Items referred to participants’ perceptions of the availability of the direct supervisors to help when needed (”Can you count on your direct boss when you come across difficulties in your work?”). The scale had excellent reliability (α = 0.90).

*Participation* was measured with three items on a 4-point response scale from 0 = “never” to 3 = “always” (”Can you participate in decisions affecting issues related to your work?”; “Can you consult satisfactorily with your boss about your work?”; “Can you participate in deciding what does and what does not pertain to your task?”). This scale also had good internal consistency (α = 0.77).

### Statistical Approach

#### Preliminary Analyses: Aggregation Statistics and Test for Common Method Bias

Before the aggregation of participation at the department-level, we calculated the *r*_*wg*_ index (LeBreton et al., 2003) and the intra-class correlation (ICC, Bliese, 2000) to establish if it is justified to aggregate participation to the unit level and to gauge the degree of non-independence of the satisfaction measure. *The r_*wg*_* index assesses the extent of consensus or within-unit variability inside a unit, and indices of 0.70 or higher support the “shared” nature of the variable in question (LeBreton et al., 2003). *ICC*(*1*) represents the ratio of between-group variance to the total variance, indicating the proportion of the total variance explained by group membership (Bliese, 2000).

A series of confirmatory factor analyses (CFA) were implemented to establish the discriminant validity of our constructs and assess the risk of common method bias influencing the results (Podsakoff et al., 2003). A multi-level CFA was conducted to determine the factor loadings of the participation items and the existence of a latent participation construct on both levels.

#### Hypotheses Testing

We tested the proposed model through Hierarchical Linear Modeling using Maximum Likelihood estimation with robust standard errors in MPlus (Muthén and Muthén, 2007). In the first step, we ran a null model assessing the variability of satisfaction imposed by unit membership. In the next step, we added the L1 predictors (PCI and supervisor support), followed by the L1 interaction term, to assess the moderating effect of supervisor support. In the fourth step, we allowed the slopes of the relationship between PCI and satisfaction to vary randomly. In the fifth step, we tested a means as outcomes model using the L2 variable (participation climate) to predict the intercept of satisfaction. In the final step, we tested the cross-level moderating effect of participative climate by regressing the PCI – satisfaction slope on the L2 predictor. For a detailed analysis of the cross-level interaction, we performed simple slopes tests using Preacher’s online tool (Preacher et al., 2006).

We centered the L2 predictor around the grand mean and the L1 predictors around the group mean. After each step, we calculated pseudo-*R*^2^ on the total, within and between variance, tested the improvement in model fit using the Bayesian Information Criteria (BIC), and hand-calculated the chi-square difference test using Satorra-Bentler scaled chi-square based on the log-likelihood (Satorra and Bentler, 2010), which is the appropriate difference test when using the MLR estimator in Mplus.

## Results

Means, standard deviations, scale reliabilities, and Pearson correlations for key study variables are summarized in Table 1.

**TABLE 1:** Correlations, reliabilities, and descriptive statistics for key study variables.

	*M* (*SD*)	α	Satisfaction (L1)	Supervisor support (L1)	Participation (L1)	Participative climate (L2)
Perceived customer incivility (L1)	0.86 (0.47)	0.76	–0.28**	–0.24**	–0.12**	–0.077*
Satisfaction (L1)	4.22 (1.22)	0.72		0.34**	0.31**	0.18**
Supervisor support (L1)	2.09 (.80)	0.90			0.55**	0.38**
Participation (L1)	1.57 (.65)	0.77				0.47**
Participative climate (L2)	1.57 (.32)					

N = 934. **p < 0.001, *p < 0.05. L1, level 1; L2, level 2.

The *ICC*(*1*) for satisfaction had a value of 0.099. Hence, about 10% of the variance in satisfaction can be explained by department-level differences, which justifies the multi-level analysis of our data. The mean *r*_*wg*(_*_*j*_*_)_ index for our measure of participation was 0.88, and the *ICC*(*1*) was 0.11, which indicates satisfactory inter-rater agreement and dependence on unit membership to aggregate participation to the department level (LeBreton et al., 2003). The multi-level CFA also supported this decision. The model which specified a latent factor of participation on both levels had a significantly better fit [χ^2^(3) = 12.54; CFI = 1.00; TLI = 1.00; RMSEA = 0.00; SRMR_within_ = 0.00; SRMR_between_ = 0.003], than the one in which the items loaded only on a within-cluster factor [χ^2^(3) = 45.394; CFI = 0.93; TLI = 0.87; RMSEA = 0.12; SRMR_within_ = 0.02; SRMR_between_ = 0.57]. Factor loadings ranged from 0.43 to 0.74 on the within level (*r*^2^ from 0.29 to 0.40), and from 0.20 to 0.24 on the between level (*r*^2^ from 0.58 to 0.9).

Further, tackling the issue of common method bias, the expected four-factor solution displayed the best fit to the data [χ^2^(84) = 462.35; CFI = 0.93; TLI = 0.92; RMSEA = 0.06; SRMR = 0.06]. A 3-factor model, with supervisor support and participation loading on the same factor, yielded a significantly worse fit [χ^2^(87) = 808.04; CFI = 0.87; TLI = 0.85; RMSEA = 0.09; SRMR = 0.06]. The one-factor solution demonstrated an inacceptable fit to the data [χ^2^(90) = 2460.05; CFI = 0.58; TLI = 0.51; RMSEA = 0.17; SRMR = 0.14], providing evidence that correlations are not driven purely by method bias (Podsakoff et al., 2003).

Table 2 reports the results of hierarchical linear regression in each step of model building. In terms of model fit, we observed a progressive decrease in BIC values, and a significant chi-squared difference between models, indicating that model fit improved by each step in the analysis.

**TABLE 2:** Results of multi-level analysis.

	Model					
	Null model (step 1)	L1 main effects (step2)	L1 interaction (step 3)	Random slope (step 4)	L2 main effect (step 5)	Cross-level interaction (Step 6)
**Level 1**						
Intercept (γ_00_)	4.24*** (0.05)	4.24*** (0.05)	4.27*** (0.05)	4.27*** (0.05)	4.26*** (0.05)	4.25*** (0.05)
PCI (γ_10_)		–0.48*** **(0.12)**	–0.45*** (0.11)	–0.45*** (0.11)	–0.47*** (0.11)	–0.45*** (0.11)
Supervisor support (γ_20_)		0.44*** **(0.06)**	0.44*** (0.06)	0.43*** (0.06)	0.43*** (0.06)	0.43*** (0.06)
PCI*Supervisor support (γ_30_)			0.50** **(0.17)**	0.42** (0.01)	0.43** (0.17)	0.41** (0.16)
**Level 2**						
Participative climate (γ_01_)					0.56*** **(0.16)**	0.66*** (0.17)
**Cross-level interaction**						
Participative climate (γ_11_)						0.98** **(0.34)**
**Variance components**						
Within-group (L1) (ε*_*ij*_*)	1.34*** (0.12)	1.16*** (0.09)	1.14*** (0.09)	1.04*** (0.09)	1.05*** (0.09)	1.05*** (0.09)
Intercept (L2) (μ_0_*_*j*_*)	0.14** (0.05)	0.16** (0.05)	0.15** (0.05)	0.17*** (0.05)	0.12*** (0.03)	0.12*** (0.04)
Slope (L2) (μ_1_*_*j*_*)				0.48*** **(0.14)**	0.45*** (0.13)	0.40** (0.01)
Intercept-slope (L2) covariance				0.18*** **(0.05)**	0.13** (0.04)	0.12** (0.04)
Bayesian (BIC)	3,007.09	2,891.82	2,878.31	2,855.41	2,847.03	2,843.32
ΔBIC		–115.26	–13.51	–22.9	–8.38	–3.71
–2LL (df)		78.77 (2) ***	8.37 (1)**	120.8 (2)***	14.17 (1)**	8.19 (1)**
Number of free parameters	3	5	6	8	9	10
Pseudo *R*^2^ total		0.109 (10.9%)	0.021 (2.1%)		0.034 (3.4%)	0.005 (0.5%)
Pseudo *R*^2^ within		0.136 (13.6%)	0.015 (1.5%)			
Pseudo *R*^2^ between					0.284 (28.4%)	**–**

L1, level 1; L2, level 2; Robust standard errors of estimates are in parentheses. ***Significant at, or below *p* ≤ 001. **Significant below *p* ≤ 01; All presented coefficients are unstandardized. New estimates derived from each step in the analysis have been bolded.

Results show that PCI significantly and negatively predicts satisfaction (γ_10_ = –0.48, *p* < 0.001); hence, the first hypothesis (H1) gained support. Moreover, supervisor support positively predicted satisfaction (γ_20_ = 0.44, *p* < 0.001), and the two L1 predictors together explained a considerable amount of 10.9% of the total variance in satisfaction (pseudo-*R*^2^ = 0.109).

**FIGURE 2:**
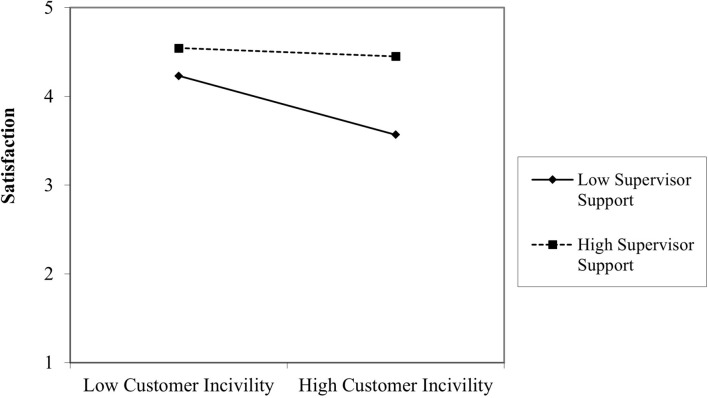
Level 1 interaction between perceived customer incivility and supervisor support.

The second hypothesis (H2) referred to the buffering effect of supervisor support in the relationship between PCI and satisfaction. The results show that the L1 interaction term is a significant positive predictor of satisfaction (γ_30_ = 0.50, *p* = 0.004), supporting H2. In other words, employees are satisfied with their job even in the case of frequent interactions with demanding clients when they perceive that their supervisor is offering sufficient support. The simple slopes analysis depicted in Figure 2 showed that at lower levels of supervisor support (1 SD below the mean), PCI predicts satisfaction negatively (β = –0.79, *p* < 0.001). However, the relationship becomes non-significant when supervisor support is high (1 SD above the mean; β = –0.10, *p* = 0.45).

Further, we found a significant variation from one work unit to another of the PCI – satisfaction slope (μ_1*j*_ = 0.48, *p* = 0.001), which suggests that the relationship between PCI and satisfaction differs among work units. Also, there was a positive covariance of the random intercept with slope, meaning that departments with higher intercepts also show a stronger relationship between PCI and satisfaction.

The third hypothesis (H3) postulated a significant direct cross-level effect of participation climate on satisfaction. The data confirm this assertion, showing that higher levels of participation climate at the department level indicate higher intercepts of satisfaction (γ_01_ = 0.56, *p* < 0.001). Not surprisingly, at the between level, the most variance (28.4%) was explained by the L2 predictor.

**FIGURE 3:**
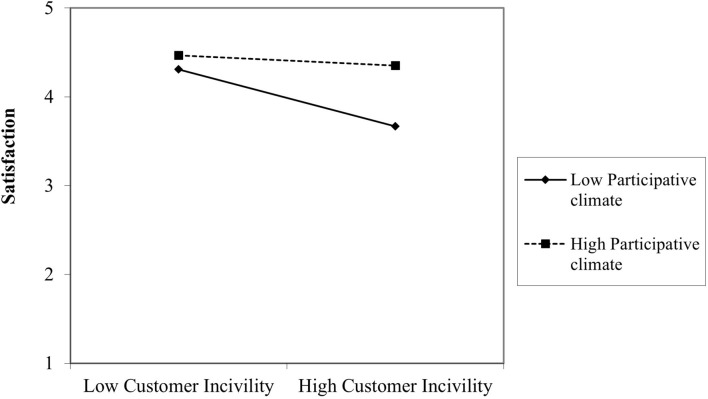
Cross-level moderating effects of participative climate.

Furthermore, the L2 variable significantly predicted the slope (γ_11_ = 0.98, *p* = 0.004), which is in line with the fourth hypothesis (H4), showing a cross-level moderating effect of participative climate (Figure 3). Thus, participative climate predicted the variability in the relationships between PCI and satisfaction among departments.^1^ The simple slope analysis showed that, at low levels of participative climate (1 SD below the mean), PCI predicts satisfaction negatively (β = –0.76, *p* = 0.000), and this relationship becomes non-significant at high levels of participative climate (+1 SD above the mean; β = –0.13, *p* = 0.286). This means that in work units where participative climate is higher, interactions with difficult clients do not relate significantly to satisfaction anymore.

## Discussion

The present study investigated the effects of perceived customer incivility (PCI) on front-line employees’ job satisfaction. We had the primary objective to identify theory-driven but practical resources from different organizational levels that can be useful for managers and organizations in helping front-line employees face difficult customers while still holding positive attitudes toward their jobs. To reach this objective, we built on widely used and influential theories in the organizational research domain: JD-R theory (Bakker and Demerouti, 2017), COR theory (Hobfoll, 2011), and SDT (Gagné and Deci, 2005), to construct a multi-level model in which individual-level resources (supervisor support), and shared resources at the work-unit level (participative climate), moderate the adverse effects of PCI on job satisfaction.

In line with our first hypotheses, results from employees in a large clothing shop chain suggest that PCI is a job demand that significantly and adversely impacts job satisfaction. According to JD-R theory (Bakker and Demerouti, 2017), employees go through an energy-depleting process when faced with sustained demands such as dealing with uncivil customers, which in turn affects outcomes like performance and work attitudes. Aligned with this theoretical reasoning and with our results, other cross-sectional studies have indicated that customer incivility is a pervasive demand in industries that imply frequent interactions with customers (Boukis et al., 2020) because it is not only directly predictive of exhaustion (Koopmann et al., 2015) but also generating more demands (van Jaarsveld et al., 2010). As employees become exhausted by customers, they also get unhappy and dissatisfied by their jobs (Alola et al., 2019). Two underlying mechanisms have been discussed in the literature for the negative effects of this demand. According to COR theory, on the one hand, front-line employees risk losing more of their resources while trying to create a welcoming environment, control their emotions and be pleasant in delivering service to uncivil customers (Hobfoll et al., 2018; Boukis et al., 2020), and these resource loss cycles are exhausting and dissatisfying for them (Alola et al., 2019). On the other hand, interactions with such customers can thwart the basic psychological needs (Gagné and Deci, 2005) of competence (i.e., feeling like a failure at ones’ job because of unfair complaints or hurtful comments at ones’ service) and autonomy (i.e., being forced to suppress emotions and be kind to another person because of the unbalanced customer-employee relationship where the customer has more power) (Koopmann et al., 2015; Chen et al., 2021). Chen et al. (2021) have demonstrated this in a longitudinal study among real estate agents, showing that customer mistreatment predicted reduced satisfaction of the needs for competence and autonomy 3 weeks later and lower job satisfaction and supervisor-rated performance 6 weeks later.

Therefore, we further hypothesized that resources in the work environment could decrease the effects of PCI and looked for resources that, according to theory, would substitute or restore the lost resources or satisfy basic needs when they are thwarted. In support of this, our data showed that supportive supervisors could have a buffering role, diminishing the negative relationship between PCI and job satisfaction. This is explained by the JD-R theory (Bakker and Demerouti, 2017), stating that job resources particularly lead to positive employee outcomes in the presence of high job demands, and employee well-being can be sustained when there are available resources to balance out the effects of demands. This proposition is consistent with COR theories’ assertion that resource gain becomes salient after resource loss (Hobfoll et al., 2018), and employees will draw on available resources provided by a supportive supervisor (e.g., guidance, training, actual help in addressing customers, and emotional support) to replenish lost resources and cope with the situation. Supervisors who offer empowering support to front-line employees can mitigate the negative impact of customer incivility and maintain psychological well-being among employees by restoring lost resources (Boukis et al., 2020). These resources are potentially fulfilling the needs for competence and autonomy (Gagné and Deci, 2005; Deci et al., 2017), which are thwarted by negative interactions with customers (Chen et al., 2021). Employees can maintain their sense of competence, and they even provide extra-role customer service toward unfriendly customers when they feel supported by their supervisor (Han et al., 2016). Building on a solid research base documenting the buffering role of support between various stress factors and employee health outcomes (Lecca et al., 2020), our results suggest that supportive supervisory behaviors can also preserve employees’ satisfaction with their jobs, regardless of being exposed to uncivil customers. Similar to our results, Han et al. (2016) have presented multi-level moderating effects of organizational and supervisory support in the employee-level relationship between customer incivility and burnout. Our study adds to this knowledge by demonstrating that supervisor support cannot only protect mental health and well-being (Han et al., 2016; Zhu et al., 2019; Lecca et al., 2020) but can also maintain positive attitudes toward work, even when it includes handling unpleasant interactions with clients.

Furthermore, as hypothesized, this study highlights the importance of considering higher-level job resources in predicting positive employee outcomes. Our results indicated that individual reports of participatory opportunities and actions aggregate within departments to a significant level, indicating that the individual resource of participation becomes a collectively understood resource shared at the department level (Kozlowski and Klein, 2000; Füllemann et al., 2016). Participative climate has positively predicted job satisfaction and explained a considerable amount of variance in satisfaction at the department level. This beneficial effect of workplace climate built around employee involvement and inclusion in decision-making replicates previous findings by Bosak et al. (2017) regarding the positive impact of employee involvement climate on job satisfaction. The most important finding of this study resides in the cross-level buffer effect of participative climate, reducing the adverse effect of PCI on job satisfaction. The shared understanding of the possibility and importance of participating in decisions is preserving employees’ satisfaction, even when interacting with unfriendly and rude customers (Han et al., 2016; Chen et al., 2021). In the COR theory perspective, participative climate constitutes a resource caravan passageway, protecting employees’ resources and generating other resources that travel in caravans (Hobfoll, 2011). Resources and their positive impact can crossover among employees who share the same environment and ultimately offer a pool of necessary resources to face difficult situations (Hobfoll et al., 2018). Placing this finding in the JD-R theory, participative climate is a resource that can foster growth, development and can motivate employees (Bakker and Demerouti, 2017) to offer better solutions and services to meet this challenge, which in turn drives performance and satisfaction (Pettijohn et al., 2007), by satisfying basic needs for competence and autonomy (Chen et al., 2021). Participation climate can be interpreted as a specific type of control over the work environment, and even employees who do not frequently participate directly can benefit from it (Weber et al., 2020). While supervisor support is a resource that can help deal with actual customers when needed, participative climate seems to be more future-oriented toward decisions and policies that are implemented to handle such interactions. Our data show a positive correlation between supervisor support and participative climate, which can signal that more supportive supervisors also create more opportunities to participate or that supervisors in such climates are perceived as more supportive. According to previous evidence, leaders can shape and influence the way subordinates perceive organizational climate (Cuadra-Peralta et al., 2017), suggesting that the two types of resources may indeed come in caravans, as COR theory describes it.

### Theoretical Implications

Individual resources exist in interaction with environmental conditions, and organizations play a significant role in the process of creating or blocking resources (Hobfoll et al., 2018). However, given the methodological challenges that multi-level research imposes, scholars often miss higher levels of analysis, and research incorporating climate with individual-level stressors is lacking (Jex et al., 2014) due to the field’s disproportionate emphasis on the individual level (Bakker and Demerouti, 2018; Hobfoll et al., 2018). Hence, the main theoretical advancement of the current study resides in the extension of the JD-R theory to investigate individual and shared group-level resources simultaneously (Nielsen et al., 2017). The model captures the negative effect of the demand and the interaction between the demand and individual well-being, which have been thoroughly investigated in past research (Bakker and Demerouti, 2018). However, our research also establishes participative climate as an essential boundary condition for these effects and as a shared resource that can have a moderating role even after the effects of direct supervisor support have been taken into account. By building a theoretical model that goes beyond individual experiences, our study, alongside recently accumulating research (Füllemann et al., 2016; Pap et al., 2020), suggest that shared perceptions and experiences in the workplace have a significant role in defining available resources and employee well-being (Schaufeli and Taris, 2014) beyond the individually offered, hands-on, practical resources that research has traditionally focused on. Climate seems to have the role of a resource caravan passageway described in COR theory (Hobfoll et al., 2018), protecting employees from resource depletion and fostering positive outcomes by being an organization or department-level source for generating other resources and protecting the ones that employees possess (Hobfoll, 2011).

### Managerial Implications

The present study identifies resources on different but complementary organizational levels, which can aid retail organizations in developing targeted interventions to reduce the negative impact of customer incivility among employees (Nielsen et al., 2017). We provide theoretically and empirically supported results, suggesting that training and encouraging leaders to offer support individually, alongside implementing HRM practices that create a participative climate among employees, can significantly reduce the effects of PCI and increase job satisfaction (Hu et al., 2018). Boukis et al. (2020) urge leaders to focus their efforts on supporting front-line employees struggling with customer verbal aggression in an empowering leadership style, which entails offering employees high guidance and training but also extensive autonomy in handling customers. Organizational participation can take various practical forms: providing information to employees, offering space to express attitudes toward this information, giving voice in decision making, involving employees in important discussions, and taking their opinions into account, veto rights, sharing power among management and employees, and importantly, leaving the freedom to decide to participate or not, to the employee (Wegge et al., 2010). For example, managers can implement Quality Circles, a participative technique that allows employees to have input into issues at work (Pereira and Osburn, 2007). These opportunities must occur frequently, and employees must be aware that they can participate in decisions regarding demanding customers if the goal is to form a climate in which employees understand that participation is an available and essential resource (Pereira and Osburn, 2007; Weber et al., 2020).

### Strengths, Limitations, and Future Research

The present study has some notable strengths, but as any study, it also faces limitations. While we discussed the motivational potential of job resources and the resource depleting and emotionally exhausting effect of job demands to build the theoretical arguments, these were constructed based on previous research and the postulates of COR, SDT, and JD-R theories, and the underlying mechanisms were not explicitly tested in our model. Research on JD-R theory has frequently relied on other theories to provide explanations for underlying psychological processes (Schaufeli and Taris, 2014). A fully supported integration can happen, however, only if future research also empirically tests the postulated mechanisms, including need satisfaction, energy depletion, and exhaustion alongside other resources that are subsequently generated by the postulated resource caravan passageway, as mediators.

The main strength of our study resides in the multi-level design, which takes into account the inherently nested structure of organizational phenomena and tackles the issue of non-independence of observations that can potentially bias estimates (Raudenbush and Bryk, 2002). However, a challenge in multi-level models is to find a large enough sample to reach predicting power at the higher levels. In this study, there is a relatively large sample size on both levels. Still, all departments are nested in one organization, which increases internal validity by eliminating potential differences regarding organizational procedures and rules that employees follow in different organizations when they face uncivil customers. However, the gain in internal validity inevitably comes with a cost in terms of external validity and generalizability of results, which warrants caution in applying our findings to other types of organizations and work contexts beyond the retail industry and shop workers.

Another limitation that warrants discussion ties back to the cross-sectional and self-reported nature of the data, limiting causal inference. Still, in a research domain where obtaining an adequate sample size on the higher levels is a challenge, longitudinal and experimental designs are challenging to implement, especially when the goal is to collect real-life field data from employees, as we did in this study. Second, the participation climate measure can spur discussions regarding the level that the construct resides on. For the most part, there is no clear consensus regarding the level of the participation construct, nor explicit discussions about whether it can most accurately be observed and measured (Kozlowski and Klein, 2000). What we assert is that a formal structure and work practices need to be in place to encourage employees to participate, but based on the emergence literature, without the individual influence that the worker exerts in decisions and the communication processes through which this resource is shared among co-workers, participation climate is unlikely to form (Kozlowski et al., 2013). This close interplay between top-down and bottom-up processes in organizations is complicated to capture and study. Some researchers might justly underline that measuring individual perceptions of participation and aggregating them to the unit level does not entirely capture the climate construct. However, the emergence literature is clear about the assertion that shared unit properties like climate can be constructed by composition models from data that is collected at the individual level. Sharedness within the unit can be evaluated through assessing within-, and between-unit variance and reliability (Kozlowski and Klein, 2000), which we carefully considered in this research. Future multi-level studies can better capture climate by more complex measures and composition models (i.e., referent-shift consensus models; Chan, 1998).

## Conclusion

This article showed that offering supervisor support and creating a work-unit climate that encourages and values participation can aid employees in dealing with customer incivility. Most importantly, not only the one-to-one support that an employee receives from a direct supervisor can maintain job satisfaction but shared understanding that one can participate in decisions that are being made in the work unit can be a source of protective resources that help the employee cope better and be satisfied with a front-line job that exposes individuals to a considerable amount of perceived incivility.

## Data Availability Statement

The data analyzed in this study is subject to the following licenses/restrictions: The raw data supporting the conclusion of this article is protected by GDPR and other data privacy reglementations and legislations in Belgium that apply to the Monitoring and Statistical Consulting Company which collected the data. Requests to access these datasets should be directed to ZP, zselyke.pap@e-uvt.ro or GN, Guy.Notelaers@uib.no.

## Ethics Statement

Ethical review and approval was not required for the study on human participants in accordance with the local legislation and institutional requirements. The patients/participants provided their written informed consent to participate in this study.

## Author Contributions

ZP, DV, and GN contributed to the design, choice of theories, and elaboration of hypotheses. GN collected and cleaned the data. DV contributed to the construction of arguments and coordinated the writing process. ZP did the analyses and produced the manuscript. Hence, all authors contributed to the article and approved the submitted version.

## Conflict of Interest

The authors declare that the research was conducted in the absence of any commercial or financial relationships that could be construed as a potential conflict of interest.

## Publisher’s Note

All claims expressed in this article are solely those of the authors and do not necessarily represent those of their affiliated organizations, or those of the publisher, the editors and the reviewers. Any product that may be evaluated in this article, or claim that may be made by its manufacturer, is not guaranteed or endorsed by the publisher.

## Footnotes

1 As additional, post-hoc analysis, we also tested a model in which we controlled for the effect of L1 participation. Our expectation was that if participation climate has a robust and unique effect that is differentiable from the L1 measure, the results should not change in this model. The results remained stable, all effects remained significant, hence we concluded that the L2 effect is robust enough, even when the L1 participation is modeled at the employee level.
